# Is there a relationship between personality and choice of nursing specialty: an integrative literature review

**DOI:** 10.1186/s12912-014-0040-z

**Published:** 2014-11-28

**Authors:** Belinda Kennedy, Kate Curtis, Donna Waters

**Affiliations:** Department of Trauma, St George Hospital, Gray St, Kogarah, Sydney, 2217 Australia; Sydney Nursing School, University of Sydney, Mallet St, Camperdown, Australia

**Keywords:** Personality characteristics, Nurses, Specialty, Retention, Stress, Burnout

## Abstract

**Background:**

Personality is deemed to play a part in an individual’s choice of work, with individuals’ preferencing a profession or field of work that will satisfy their personal needs. There is limited research exploring the personality characteristics of nurses within clearly defined nursing specialty areas. Retaining nurses within specialty areas has workforce implications when vacancies are unable to be filled by appropriately experienced staff.

The aim of the review was to determine the current state of knowledge regarding the personality profiles of nurses in specialty areas of nursing practice.

**Methods:**

An integrative literature review was undertaken.

Five electronic databases were searched using personality and nursing based keywords. No date limit or research design restriction was applied. Rigorous screening and quality appraisal was undertaken considering the research design, methods and limitations of each manuscript.

**Results:**

A review of the 13 included articles demonstrated some variability in the personality characteristics of the nursing specialty groups studied. A relationship was identified between personality characteristics and levels of nursing stress and burnout.

**Conclusion:**

There is some evidence to suggest a relationship between personality characteristics and nursing specialty choice, burnout and job satisfaction. The published literature is limited and the effect of personality on retention is not well established.

**Electronic supplementary material:**

The online version of this article (doi:10.1186/s12912-014-0040-z) contains supplementary material, which is available to authorized users.

## Background

The nursing workforce is ageing, leading to a global shortage of experienced nurses [1-3]. At the same time there is increasing demand within the health care system [2]. Problems with retaining nurses in the workforce are not new or isolated to one particular field and factors contributing to retention are multifaceted. These include stress and burnout caused by the type of nursing care [4,5], as well as workplace related issues such as patient ratios and increased workloads, career progression and pay [6]. Poor retention has particular implications for specialty areas where vacancies must be filled by staff with experience in the area, for example intensive care [7-9]. As a consequence of advances in technology and health care, it takes significant resources to train nurses to function as experts in their specialty [10]. For example, it can take up to two years for an emergency nurse to attain the requisite knowledge base and skills to progress to triage training, for oncology nurses to perform plasmapheresis or for intensive care nurses to perform extracorporeal membrane oxygenation.

Personality is believed to play a role in career choice [11,12] and it is postulated that an individual will seek a profession or role that will provide personal satisfaction and meet their personal needs [13,14]. As early as 1927, research exploring personality in nursing highlighted differences in personality between nurses and other college educated women, as well as paediatric and general nurses [15]. More recently, McPhail [16] explored the relationship between personality and four domains within nursing: direct care, administration, teaching and research. This study found that nurses are attracted to a particular area of nursing as a result of personality, and clusters of similar personalities were able to be identified in these different roles [16]. With these findings and research that has identified differences in personality within other health professional groups [17,18], it is reasonable to suggest that the personality characteristics of an individual may influence their choice of nursing specialty area and influence the time an individual spends working within a specialty. However, there is limited research evaluating the personality of qualified nurses in defined clinical specialties.

Personality testing enables the identification of an individual’s personality characteristics. These characteristics can inform how an individual is likely to respond or cope when exposed to different situations [19]. Personality testing was used in United States (US) army recruitment during World War I in an attempt to predict those who may be predisposed to develop psychological disorders [20]. Although controversial, use of personality testing in recruitment has been reported to be used in up to 20% of US companies [21].

It is well-recognised that personality has an influence on the way an individual interacts and deals with the outside world, and in turn, influences their ability to cope and deal with stressful situations [22]. Personality characteristics have the potential to provide an explanation as to why some individuals manage to deal with stress and continue to function effectively, while for others, the same situation may cause major disruption to their physical and mental wellbeing [22]. Stress and burnout are recognised as a workforce issue for nurses and have been found to impact upon staff retention. Previous studies have identified a relationship between hardy individuals and decreased levels of stress and/or burnout [23-25]. Hardiness theoretically functions as a moderator, assisting individuals in dealing with stressful events [24,26]. Hardiness is comprised of three personality dimensions: commitment, control and challenge. Hardy individuals’ possess a greater sense of control over their environment, a situation that assists the individual to deal with stressful events and situations [25,26]. Hardiness has been linked to stress and burnout, and burnout has been shown to impact upon job satisfaction and hence influence nurses’ decisions to remain within the workforce [27], therefore, hardiness is included as a personality trait in this review.

To determine the relevance of personality testing in nursing, any link between the personality characteristics of an individual and specialty practice choice along with other factors such as stress, burnout and job satisfaction must first be established.

The aim of the integrative review is to determine the current state of knowledge regarding the personality profiles of registered nurses according to clinical specialty areas of nursing practice.

## Methods

A scoping review was performed to determine what literature review strategy was best suited to answer the aim of the study. The quality and nature of papers found in the initial search was inappropriate for a systematic literature review method, and more suited to an integrative approach. An integrative literature review method allows for the incorporation of different types of literature and a broad range of methodologies [28]. Additionally it permits the integration of theoretical work around the research subject [29].

The population selected for the review was qualified nurses working within a defined clinical specialty area. Studies that explored personality profiles/type or hardiness within this population group were targeted. The review process involved a search of current literature, quality evaluation of recovered data and synthesis of findings [29]. A keyword search was performed using five electronic databases and hand searching of references. The electronic databases CINAHL, Medline, PsycINFO, Proquest 5000 and WORKLIT were searched combining keywords, for example ‘personality’, ‘nursing’ and ‘career choice’ (Keywords for database search). The ‘emergency nursing’ specialty was the initial area of interest, however, searches using these keywords yielded few results. Search terms were then broadened, limited to English language and further searches performed as detailed in Keywords for database search. No date limit or research design restriction was applied as a result of limited results with the initial searches.

### Keywords for database search

- **Personality Keywords (combined with OR)**
  - Personality
  - Personality style
  - Personality trait
  - Personality type#
  - Personality characteristic
  - Personality assessment
- **Search terms for CINAHL (Combined with Personality keywords)**
  - AND emergency nurse* OR critical care nurse* OR intensive care nurse*
  - AND career choice OR occupational choice
  - AND nurs*
- **Medline, Psychinfo, Proquest 5000 and Worklit search terms (Combined with personality keywords)**
  - AND career choice OR occupational choice AND nurs*
  - AND career choice OR occupational choice AND specialty ^**#**^
  **#** terms used in Medline only. * asterisk used on the end of search terms provides both singular and pleural forms of the word in search results.

**Figure 1:**
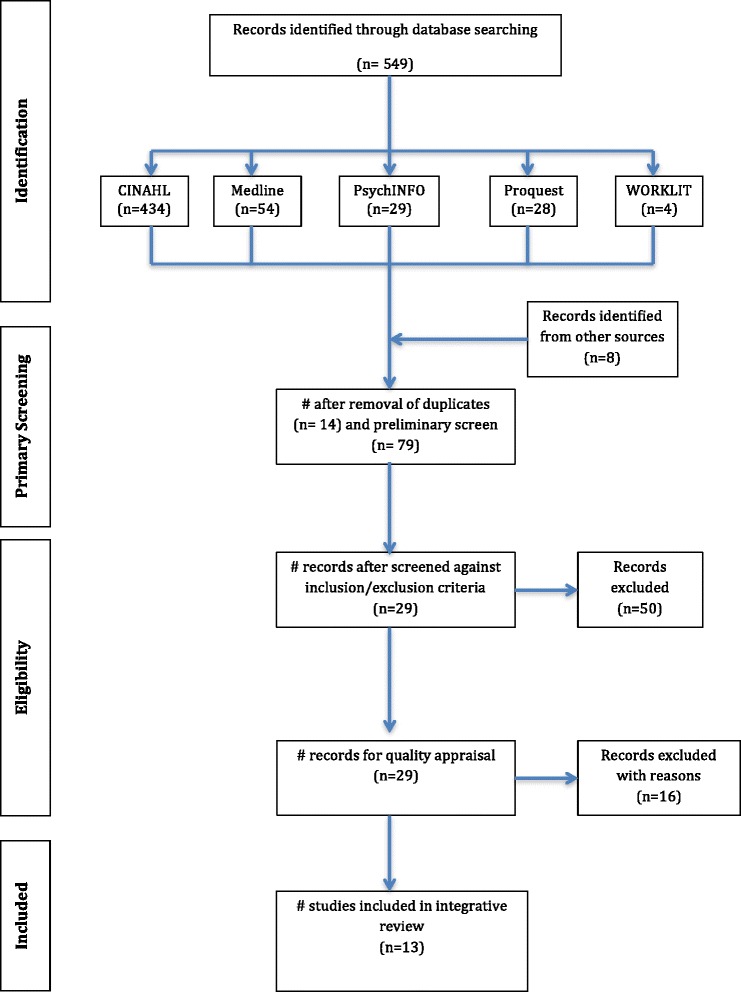
**PRISMA flow diagram.**

The search and screen process of retrieved literature followed the PRISMA framework [30] summarised in the Figure 1. While the PRISMA framework was designed for use in systematic reviews and meta-analysis [30], it has been used in this integrative review to demonstrate the steps undertaken to determine the final papers included in the review.

The initial search identified 549 published papers. Papers were considered relevant to the review if they presented research related to the study of personality in nurses. Titles of the papers were screened and those deemed not relevant were removed (n = 464). Following relevance checking, duplicates were removed (n = 14) resulting in 71 articles. The reference lists of these articles were hand searched, identifying a further 8 papers. The abstracts of these 79 articles were then independently peer reviewed by each author using pre-determined inclusion/exclusion criteria. Full text copies were retrieved when abstracts were not available. Papers were included in the final review if they were original research with a clear research design and explored hardiness or personality among nurses working in defined nursing specialty areas.

Papers were excluded if they were not original research or failed to report separate results for qualified nurses when nursing students were included in the sample. Nursing students do not necessarily choose their area of clinical placement, therefore inclusion of their personality characteristics has the potential to skew findings within specialties. Nine PhD theses that met inclusion criteria were identified by the search, however, further database searching failed to identify any subsequent papers published by the authors. Only one thesis was available in full text and the ability to obtain other theses identified by the database search was beyond the resources of this study. Hence all theses were excluded. Differences of opinion or uncertainty between authors regarding the relevance, inclusion or exclusion of an article were resolved by discussion.

Following the screening process, 29 papers were obtained in full text and reviewed independently by all three authors using a critical appraisal tool adapted from the quantitative appraisal guides of Polit and Beck [31]. A total of 16 papers were excluded: 13 primarily for reporting mixed populations (for example student nurses and qualified nurses or mixed nursing specialties) where results could not be extrapolated to discrete clinical groups. The remaining papers were excluded because one related to nurses working as nurse practitioners and not within a single clinical specialty area; one provided insufficient information within the report to enable critical analysis and for the other, only an abstract was published.

## Results

The systematic search and quality appraisal of published and unpublished literature resulted in 13 articles of sufficient quality for inclusion in the integrative literature review (see Additional file 1). The overall quality of research exploring personality in nursing career choice was poor and the sample sizes in most studies were small. The final studies included in the review are predominantly from the US and published between 1965 and 2010.

A range of tools were used to measure personality in the studies (see Additional file 1). The most common were the Personal Style Inventory and the Myers Briggs Type Indicator. Both tools use self-report to measure four independent dimensions derived from Carl Jung’s Dimensions of Personality Theory: introversion – extraversion; sensing – intuition; thinking – feeling; and judging – perception. These four diametric dimensions attempt to explain the way individuals perceive and then judge perceptions, with combinations resulting in 16 possible personality types [11,32,33]. Other personality tools reported in the final 13 papers were those based upon Henry Murray’s psychological needs (for example the Edwards Personal Preference Schedule) [34,35] and Raymond Cattell’s (16 Personality Factors) theory of personality [36,37]. The personality factors in Raymond Cattell’s theory have been demonstrated to correspond closely to the five factor model (FFM) of personality which is the most validated and widely accepted theory for personality research [38,39]. One study used the NEO-PI-R, which is regarded as a broad, comprehensive measure of normal personality measuring the five domains of personality [4,40].

Four distinct areas of study were identified from the synthesis of collated literature from the integrative literature review:

1. Exploration of personality characteristics within a nursing specialty
2. Comparing personality characteristics between different specialty areas within nursing
3. The role of personality in stress and burnout
4. Relationship between personality and job satisfaction

The papers were clearly identifiable within one or more of the above themes. The findings of the review are discussed within each of these themes below.

### Exploration of personality characteristics within a nursing specialty

Atkins and Piazza [41], Bean and Holcombe [11], Gambles et al. [36], Levine et al. [37] and Lewis et al. [5] explored personality within the oncology, emergency, intensive care and renal nursing specialties. Cattell’s 16 Personality Factors (16PF), the Personal Style Inventory (PSI), the Hogan Champagne Preference Survey and the Myers Briggs Type Indicator (MBTI) were used to measure personality in these studies (see Additional file 1). Overall, the study designs were not sufficiently rigorous to make firm conclusions, but do suggest some variation in the personality characteristics of the groups that may be attributed to the different specialty area of nursing. Studies profiling the personality of emergency [41], oncology [11] and renal [5] nurses have found that a larger proportion of their sample demonstrated the personality trait of *introversion*. Introverts are described as being task orientated, independent and diligent [11], preferring to work alone and maintain control over their environment [11,41].

Similarly, Gambles et al. [36] and Levine et al. [37] used Cattell’s 16 PF to study the personality of oncology and critical care nurses. There were many similarities between the two groups in the 16 personality factors measured. While the scores largely fell within population norms, personality factors with the greatest difference in scores were: *dominance, emotional sensitivity, suspiciousness, rebelliousness* and *self-sufficiency*. Critical care nurses scored higher on *dominance, rebelliousness* and *self-sufficiency* and lower on *emotional sensitivity* and *imagination*. Cancer and palliative care nurses scored high on *emotional sensitivity* [36], the one factor outside the population norm. High scores on this factor are considered to be associated with individuals who are aware of their own feelings, compassionate and understanding [36]. Critical care nurses scored highly on *self sufficiency* [37]. Higher scores on this trait are generally indicative of individuals who prefer to be self-sufficient and have a preference to make their own decisions independently. This is in contrast to cancer nurses who scored low on *self-sufficiency*. Individuals scoring lower on *self-sufficiency* are generally more group orientated and prefer to work with others as opposed to independently [36]. While both oncology and critical care nurses scored at the extreme ends of the *self-sufficiency* scale, both scores remained within the normal range.

### Comparison of personality characteristics between different specialty areas within nursing

Almost fifty years ago, Lentz and Michaels [34] explored basic personality factors among female medical and surgical nurses using the Edwards Personal Preference Schedule (EPPS). This study reported nurses scored significantly higher than population norms for the psychological needs of *order* and *endurance*, and scored lower for the need of *dominance,* when compared to Edward’s female population norms. A large proportion of the sample consisted of nursing students, and removal of this subgroup from analysis accounted for the variance between this sample of nurses and the population norm. The differences noted were therefore attributed to the younger age of the nursing students [34]. Results were then compared to an earlier study using the same tool in a sample of mental health nurses. The study noted significant differences between medical/surgical nurses and mental health nurses on eight variables, the most significant being the psychological characteristics of *abasement, exhibition* and *introception*. It must be noted that students were included in this analysis and may have influenced the results reported [34].

In another study using the EPPS, Stauffacher and Navran [35] investigated whether personality characteristics could predict the area of professional engagement of nursing students five years post qualification. While they found no significant difference among specialty groups when looking at area of practice, they did find significant differences among nurses’ pre-experience EPPS scores on the personality variables of *achievement*, *order* and *introception*. Notably, a large number of the study sample was not employed in their preferred clinical area.

Cross and Kelly [33] compared personality and anxiety levels in intensive care (IC) nurses and medical/surgical nurses and found some similarities in personality between the two groups. While a number from both groups self-selected a preference for *introversion* and *sensing* characteristics, a significantly higher proportion of IC nurses than medical/surgical nurses self-selected as *thinkers* (65.9% vs 41.8%). Those who fall within the *thinking* dimension are known to take a more logical and objective approach in decision making, not allowing emotions to influence the process [33,41].

The results of these studies suggest that there may be differences in personality among nurses who chose to work in different specialty areas.

### The role of personality in stress and burnout

The results of studies exploring the relationship between personality, and stress and burnout are difficult to interpret and compare because different personality variables have been measured with different tools [4,5,25,42]. A study of intensive care nurses using the NEO-PI-R (Additional file 1) reported a significant negative correlation between the personality traits of *openness* and *extraversion*, and stress when dealing with patients and relatives, with no positive relationship identified between personality and workplace stress [4]. Others [33,42] have demonstrated links between hardiness (or sense of coherence) with levels of stress and/or burnout among intensive care and renal nurses.

Another study exploring the effects of hardiness among critical care nurses [25] reported significant correlations between hardiness and stress and burnout. A positive correlation was demonstrated between hardiness and burnout, nurses with greater hardiness (lower scores) experienced lower levels of burnout. However, analysis of the three dimensions of hardiness independently, demonstrated the relationship between stress and burnout was attributed to only one of the dimensions of hardiness, *commitment*. One dimension of hardiness (*control*) yielded a positive correlation with occupational stress, that is higher scores on the *control* dimension were associated with higher levels of occupational stress. In contrast, Toscano and Ponterdolph [26] reported no correlation between hardiness and burnout in a group of 100 critical care nurses. The authors themselves cite potential weakness in the methodology that may have influenced the result.

Sense of Coherence (SOC) is a concept that has been closely related to hardiness [5] and has been described to assess how an individual sees the world. Individuals with a strong SOC ‘view life as ordered, predictable and manageable’ [5]. Those with a higher SOC theoretically experience lower levels of stress and burnout, similar to those considered to be hardy individuals. Lewis [5] also found significant negative correlations between high SOC and work stress, and high SOC and personal stress.

### Relationship between personality and job satisfaction

Only one paper in this review (see Additional file 1) explored the relationship between personality and job satisfaction. Using the MBTI among a group of 923 Dutch nurse anaesthetists, Meeusen et al. [43] identified two personality dimensions, *easy going* and *orderly* that explained 3.5% of the variance and were significant predictors of job satisfaction among nurse anaesthetists. This study suggests that personality dimensions may potentially influence the level of job satisfaction among nurses.

## Discussion

This integrative review of research literature offers some evidence that personality differences are associated with nursing specialty choice, levels of job satisfaction and stress and/or burnout among nurses. Considering the array of nursing specialties, roles, practice environments and the pressures on nursing recruitment and retention, this field of research warrants further investigation.

The personality measures used in the various studies are underpinned by a variety of personality theorists, including Carl Jung, Raymond Cattell and Henry Murray; these are known as trait theorists. A personality trait is the aspect of personality that is considered to remain stable over time and situations [44]. Any change in a personality trait is gradual and generally seen as a result of maturation with age [40]. Personality traits are associated with, and may predict, the way in which one will respond in a particular context or situation and how we as individuals interact with the environment around us [45-49].

A wide range of tools and methods were used to measure personality in the final set of reviewed literature, and within those studies, different versions of the same tool were used. The majority of studies included in this review were limited by small sample sizes, compromising comparison and synthesis of the results. The level of evidence presented in these studies is insufficient to reliably generate a personality profile for any of the nurses for the specialties studied. In order to be able to make any conclusions larger studies across various specialty areas are required.

A number of papers in this review noted that a large proportion of nurses studied scored higher on the *introversion* characteristic of personality. The introversion – extraversion dimension of personality relates to the way individuals’ process information and make decisions. Individuals with high scores for the *introversion* dimension think things through, internalising thought processes in order to come to a decision. However, others reported findings more consistent with the *extraversion* characteristics of personality, that is, those who are more empathetic, act quickly and externalise thought and actions. These results may simply be reflecting the diversity of the nursing workforce, or recognising that everyone has a different way of managing information to come to a decision. A weakness of these studies is that none reported the actual scores of the study sample and hence the results were simply recorded as falling within either the introversion or extraversion dimension. Those whose scores fell within the middle of the dimension are also unidentifiable. Those who score in the middle range, referred to as ambiversion [50], possess the qualities of both dimensions, that is, they alternate between introversion and extraversion given the requirements of the situation.

*Sensing* was also noted to be common among different groups of nurses. The sensing – intuition dimension refers to the way individuals perceive the outside world. Those who possess the characteristic of *sensing* prefer evidence that is presented in a way that can be assessed and comprehended using the five senses, in contrast to those who are identified as *intuitive* who show greater interest in the underlying theory and principles of data. While both characteristics are certainly recognised as being essential for nurses, it is not surprising that the personality characteristic with highest self selection was *sensing*, given the expectation is that nurses have the ability to assess and evaluate situations using their senses in order to make sound clinical decisions in the workplace.

The studies that sought to explore differences in personality between two or more defined specialty areas reported variability between the groups on a number of personality characteristics. This suggests that not all nurses can be grouped together when considering personality but that clusters of like personality characteristics can be identified among those choosing to work within the same specialty area [33,34]. It is plausible, for example, that observed differences in *self-sufficiency* among critical care and oncology nurses may be reflective of different personalities within the specialty areas.

Critical care work environments can contribute to significant stress for nurses due to the high frequency of immediate life threatening and unpredictable situations [51]. Critical and intensive care nurses frequently work independently, managing all aspects of their one critically ill patient. Higher scores on the personality domains of *self-sufficiency* and *thinking* reported within these specialties may be consistent with a need to manage a stressful work environment.

There are a number of factors thought to contribute to stress in the nursing profession, for example, exposure to the sick and dying, caring for long term patients as well as high workload, high patient turnover and acuity, staffing demands and professional roles and expectations [4,5]. Stress and burnout are known to impact upon the retention of nursing staff [6] and personality factors such as hardiness can perhaps enable nurses to manage this stress. However, studies exploring hardiness in this review used a number of different methods and yielded conflicting results. There is insufficient evidence to draw a conclusion regarding the impact of hardiness as a personality trait on stress and/or burnout, or the impact of personality in general on stress and burnout in the nursing workforce.

The evidence related to the relationship between personality and job satisfaction is limited in this review as only one study explored this concept. The results of this study indicated that some select characteristics demonstrated a positive relationship to job satisfaction. This is most certainly an area of study that warrants its own dedicated investigation, given it is well recognised that if an individual is dissatisfied with their work role they are more likely to consider other job opportunities. The ability to stimulate and satisfy an individual, with consideration to personality, has the potential to improve job satisfaction and in turn, retention.

While personality testing, inclusive of psychometric and aptitude tests, has been reported as part of recruitment practices [21,52], there are conflicting views on their usefulness. Some believe that personality tests may discriminate against individuals due to culture or gender differences [39], that personality test results can be faked, and are not necessarily valid predictors of job performance [53]. Personality testing has the potential to play a role in the recruitment of nursing staff suited to a particular specialty, just as it has been considered in some medical fields, such as anaesthesia [17,54]. It is recognised that there is still considerable work that needs to be done in order to establish the usefulness of personality testing, including establishing valid and reliable methods of personality assessment, before it is to be incorporated in recruitment practices within the healthcare profession [55]. The suggestion is not to use personality testing to prevent people working in a particular specialty area, rather, to facilitate targeting of nurses more suited to a clinical area. Personality profile information may also be used to further explore stress/burnout and job satisfaction within defined nursing specialty areas for the purpose of improving retention. Prior to undertaking such investigation, a clear personality profile of the nurses successfully employed within a defined specialty is needed.

The studies included in this review were performed over 45 years (from 1965–2010). The nursing profession and the role of nurses has evolved considerably across all nursing specialties during this time [56,57]. If personality characteristics were to be used to inform workforce planning and improve nurse retention in specialty areas, it would be necessary to have an evidence-base that reflects the current nursing workforce. This integrative review of research literature has shown that currently, this evidence does not exist.

## Conclusion

There is some evidence of variance in personality characteristics between different nursing specialty areas, as well as associations between personality characteristics and stress, burnout and job satisfaction. However these variances occur largely within normal ranges. The investment required to train nurses to function as a “specialist” implies a benefit exists in targeting individuals potentially suited to particular specialty nursing areas to optimise retention. More robust research using tools based on the five factor model of personality is required to generate evidence for the theory that particular individuals are suited to different nursing specialties.

If employees possess the personality characteristics that are best suited to the job, this will likely result in improved workplace efficiency, job satisfaction and retention of staff. All of these factors in turn have capacity to influence the quality of care delivery. Clearly identified personality characteristics linked to stress and burnout have the potential to enable appropriate interventions and plans to be put in place to assist in improving the nurses’ ability to deal with workplace stressors. This in turn would assist to reduce staff turnover in stressful work environments.

## Additional file

Additional file 1:
**Findings of the Review.** File provides a summary of the 13 articles included in integrative review.
