# Supplementary material for: Is there a relationship between personality and choice of nursing specialty: an integrative literature review
Source: BMC Nurs. 2014 Nov 28;13:40. doi: 10.1186/s12912-014-0040-z (PMC4267136; doi:10.1186/s12912-014-0040-z)
Supplement: Additional file 1: — Findings of the Review. File provides a summary of the 13 articles included in integrative review. [file 12912_2014_40_MOESM1_ESM.pdf]

Additional File 1: Findings of the review

Authors (year)	Sample (setting)	Personality tools	Findings (personality factors in italics)
**Atkins, Piazza (1987)**	46 emergency nurses with < 2 years emergency experience (USA)	Hogan Champagne Preference Survey (also known as Personality Style Inventory)	Most common combination: *introversion, sensing, feeling* (ISF) 22%; followed by *introversion, sensing, thinking, judging* (ISTJ) 11%.<br>Most common individual characteristics: *introversion* (63%); *sensing* (61%); *feeling* (65%); *judging* (61%).
**Bean & Holcombe (1993)**	40 oncology nurses (USA)	Personal Style Inventory (PSI)	Most common combination: *introversion, sensing, feeling, judging* (ISFJ) 32%.<br>Most common individual characteristics: *introversion* (65%); *sensing* (57%); *feeling* (55%); *judging* (50%) .
**Buhler & Land (2003)**	117 intensive care nurses (Germany)	Eysenck Personality Inventory (EPI); Trier Personality Questionnary (TPQ); Scales of Control; Locus of Control and; LOGO test.	Relationship between personality and burnout (measured by three dimensions of Maslach Burnout Inventory: Emotional Exhaustion (EE); Personal Accomplishment (PA) and; Depersonalisation (D)): *fatalistic external locus of control, job-distance inability, existential frustration, extraversion* and *neuroticism* significant predictors of EE, explaining 43% of the variance (p<0.0001) in multivariate regression analysis. *Existential frustration* and *extraversion* significant predictors of PA, explaining 15% of the variance (p<0.0002). *Ability to love, extraversion* and *neuroticism* significant predictors of D, explaining 12% of the variance (p<0.0001). Relationship between *external locus of control* (a measure of hardiness) and burnout: significant +ve correlation with EE (0.27, p<0.01) and D (0.18, p<0.05).
**Burgess, Irvine & Wallymahmed (2010)**	46 intensive care nurses (UK)	NEO Personality Inventory REVISED (NEO PI-R)	Relationship between personality and stress (measured by Nurse Stress Index): significant -ve correlations between *openness* and stress *(-0.31,p<0.03)*, *extraversion* and stress *(-0.33,p<.02)* and between *conscientiousness* and workplace stressors (time management -0.34,p<0.02 and management demands -0.47,p<0.01). Relationship between personality and coping (measured by Brief COPE): significant +ve correlations between *openness* and planning (0.39,p<0.01), *openness* and reframing (0.47,p<0.01), *conscientiousness* and planning (0.40,p<0.01*), conscientiousness* and active coping (0.33, p<0.03), *agreeableness* and active coping (0.38,p<0.01) and *agreeableness* with both planning (0.36,p<0.02) and reframing (0.34,p<0.02).
**Cross & Kelly (1984)**	55 medical/ surgical & 41 intensive care nurses (Australia)	Myers Briggs Type Indicator (MBTI)	Relationship between personality and anxiety (measured by Taylor Manifest Anxiety Scale): both groups most commonly cluster in *introversion* (73%) and *sensing* (67%) quadrant (IS) incorporating ISTJ, ISFJ, ISTP, ISFP combinations. Significant correlation between *introversion* and anxiety (0.34,p<0.02) for ICU group only.
**Gambles, Wilkinson & Dissayake (2003)**	178 cancer & palliative care nurses (UK)	16 Personality Factor (16PF) (VERSION A)	6/16 primary order factors were extreme (very low or very high) or outside population norms. Higher scores on *emotional sensitivity*; *impulsivity* and *imagination*, lower scores on *self-sufficiency*, *suspiciousness* and *rebelliousness*. 2/8 second order factors were high (e*xtraversion*) or low (*tough poise)*.
**Lentz & Michaels (1965)**	384 medical/ surgical nurses (USA)	Edwards Personal Preference Schedule (EPPS)	Nurses score higher than population norms for *order, endurance, deference* (not controlled for age) and lower than norms for *dominance*. Possible differences on 10/15 personality factors when compared to a mental health nurse cohort reported by Navaran,& Stauffacher (1958).
**Levine, Wilson & Guido (1988)**	200 critical care nurses (USA)	16 Personality Factor (16PF) (FORM C)	4/16 primary order factors were extreme (very high) compared to population norms. Higher levels of *dominance*; ego strength (*leadership& conforming); self-sufficiency; controlled (socially precise).*

Additional File 1: Findings of the review (continued)

**Lewis, Bonner, Campbell, Cooper & Willard (1994)**	49 nephrology nurses (USA)	Myers Briggs Type Indicator (MBTI) FORM G	Most common combinations: INFJ* (14%), ISTJ* (12%), ESFJ* (12%).<br>Most common individual characteristics*: introversion* (55%); *intuition* (51%); *thinking* (55%); *judging* (65%). No significant relationship between personality and personal stress (measured by Perceived Stress Scale and work related stress (measured by Nurse Stress Scale). No significant relationship between personality and coping (measured by Sense of Coherence Scale and Coping Resources Inventory). No significant relationship between personality and burnout (measured by Maslach Burnout Inventory).
**Meeusen, Brown-Mahoney, Dam, Zundert, Knape (2010)**	923 anaesthetic nurses (Netherlands)	Myers Briggs Type Indicator (MBTI)	Relationship between personality and job satisfaction (measured by validated instrument): *Easy going* and *orderly* significant predictors of job satisfaction in multivariate regression analysis, but explaining only 3.5% of the variance.
**Stauffacher & Navran (1968)**	453 nursing students followed up after five years of practice (USA)	Edwards Personal Preference Schedule (EPPS)	Change in 11/15 factors on EPPS after five years. Significantly higher mean difference (MD) in *achievement (MD 2.2,p<0.001), order (MD 2.0,p<0.001) and heterosexuality* (MD 1.7,p<0.001) and significantly lower score for *abasement(MD 1.56,p<0.001).* No significant relationship between personality and actual specialty experience in first five years but significant for *achievement* (F=3.2,p<0.01) *order* (F=3.01,p<0.05) and *intraception* (F=2.99,p<0.05) when analysed by preferred specialty. High *dominance* and *exhibition* in those preferring administration, teaching and research (after five years); high *intraception* in mental health nurses across all five years.
**Topf (1989)**	100 critical care nurses (USA)	Dimensions of Hardiness:<br>1. Commitment<br>2. Control<br>3. Challenge	Relationship between hardiness and occupational stress (measured by Nurse Stress Scale): significant +ve correlation between *external locus of control* and stress (0.34,p<0.001)<br>Relationship between hardiness and burnout (measured by Maslach Burnout Inventory): only one dimension hardiness (commitment) linked to all three dimensions (EE, PA & D) of burnout.<br>No significant relationship between occupational stress and burnout.
**Toscano & Ponterdolph (1998)**	100 critical care nurses (USA)	Third Generation Hardiness Test	No significant relationship between hardiness and burnout (measured by Maslach Burnout Inventory).
